# Identification of a DNA damage repair gene‐related signature for lung squamous cell carcinoma prognosis

**DOI:** 10.1111/1759-7714.14370

**Published:** 2022-03-15

**Authors:** Bin Jia, Ting Gong, Bingsheng Sun, Zhenfa Zhang, Diansheng Zhong, Changli Wang

**Affiliations:** ^1^ Department of Lung Cancer, Tianjin Lung Cancer Center Tianjin Medical University Cancer Institute and Hospital, National Clinical Research Center for Cancer, Key Laboratory of Cancer Prevention and Therapy, Tianjin's Clinical Research Center for Cancer Tianjin China; ^2^ Department of Medical Oncology Tianjin Medical University General Hospital Tianjin China

**Keywords:** DNA repair, lung squamous cell carcinoma, prognosis, signature

## Abstract

**Background:**

DNA damage repair (DDR) plays a role in the tumorigenesis and progression of lung squamous cell carcinoma (LUSC), but the predictive value of DDR in LUSC has not been fully elucidated.

**Methods:**

The LUSC datasets were retrieved from the Cancer Genome Atlas databases. Univariate Cox regression and least absolute shrinkage and selection operator regression were integrated to identify critical genes and construct a DDR gene signature. We performed Kaplan–Meier (KM) curve to compare the overall survival (OS) between the two groups based on DDR signature and used the CIBERSORT tool to compare the immune cell composition. Further gene set enrichment analysis (GSEA) was performed on the differential expressed genes.

**Result:**

We established the DDR‐related gene signature on LUSC. KM curve showed the low‐risk group had a better prognosis than the high‐risk group in the training set (*p* = 0.022673) and the complete set (*p* = 0.003201). The area under receiver operating characteristic curve for OS was 0.98, 0.96, and 0.97 in the training dataset, testing dataset, and the complete dataset, respectively. The composition of immune cells was different between the high‐ and low‐risk group. The GSEA result suggests that genes of the patients in low‐risk group were mainly enriched in the DNA adducts; drug metabolism‐cytochrome P450, metabolism of xenobiotics by cytochrome P450.

**Conclusion:**

This study identified DDR‐associated potential biomarkers related to overall survival of LUSC and establishes the DDR‐associated gene signature.

## Graphical abstract

This study established a DNA damage repair (DDR)‐related gene signature on lung squamous cell carcinoma with the data from the Cancer Genome Atlas. The DDR signature has prognostic value, and we verify it in training group, testing group, and complete group. Moreover, we have done the Gene Ontology enrichment and gene set enrichment analysis between the high‐ and low‐risk groups defined by signature.

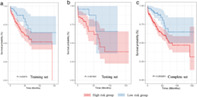

## INTRODUCTION

Lung cancer remains the precedent cause of cancer death, with ~1.8 million deaths in 2020.^1^ Around 85% of lung cancer cases correspond to non–small‐cell lung cancer (NSCLC),^2^ including lung adenocarcinomas (LUAD) and lung squamous cell carcinoma (LUSC). More than 60% of LUAD can be identified by driving mutations, epidermal growth factor receptor (EGFR), ALK, and other driver gene mutations, which significantly improved survival in patients with LUAD; however, for LUSC, accounting for one‐third of NSCLC, the driver genes such as EGFR mutations and ALK gene rearrangements are rarely detected.^3^ However, increased EGFR gene copy and protein overexpression are more common in LUSC than in LUAD,^4^ and LUSC has a high total mutation rate and significant genetic complexity.^5^ LUSC has some apparent driver gene mutations, including PIK3CA, AKT, FGFR1,^6^ but there is no related effective treatment.

Widespread intratumor heterogeneity can be detected in lung cancer for both somatic mutations and copy‐number alterations.^7^ Driver mutations that occurred later in evolution were almost always clonal or subclone and involved in chromatin modification and DNA damage response and repair.

DNA damage response is crucial to prevent the accumulation of DNA lesions and mutations that may promote carcinogenesis. It can repair the damaged DNA by one or several pathways: base excision repair (BER); homologous recombination (HR); direct repair (DR); nucleotide excision repair (NER); mismatch repair (MMR) or non‐homologous end joining (NHEJ).^8^ When DNA damage repair (DDR) pathway is impaired, cytoplasmic DNA gathers, and DNA replication pressure increases, which not only leads to genomic instability, but also induces the release of validation‐related factors and activates the innate immune response by activating the cyclic GMP‐AMP synthase‐stimulator of interferon genes (cGAS‐STING) and/or nuclear factor‐κB (NF‐κB) pathway.^9^

In this study, the Cancer Genome Atlas (TCGA) (https://portal.gdc.cancer.gov/) database was involved in investigating the relationship between DDR genes and the LUSC prognosis and finally to establish DDR‐associated prognostic signature. Here, we screened specific DDR genes that related to the prognosis of LUSC and classified the LUSC patients into high‐ and low‐risk groups based on the DDR‐related genes expression. We confirmed the classification with Kaplan–Meier (KM) curve analysis and tSNE cluster and verified the prognostic signature with multiple receiver operating characteristic (ROC) curve. In addition, we explored the distribution and composition of immune cells in the tumor microenvironment and performed Gene Ontology (GO) enrichment, and gene set enrichment analysis (GSEA) with differential expressed DDR genes between high‐ and low‐risk groups. Based on the above analysis, we demonstrate the critical role of specific DDR gene in LUSC prognosis, indicating the potential use of this signature.

## METHOD

### Data collection from TCGA and GSEA

Lung squamous cell carcinoma RNA transcriptome dataset and their relevant clinical information were retrieved from the TCGA database. Genes were grouped into protein‐coding genes based on the human genome annotation data. The list of DDR‐associated gene set was obtained from the Molecular Signatures Database (https://www.gsea-msigdb.org/gsea/msigdb).

### Identification of differentially expressed DDR genes in LUSC

We used R software (https://www.r-project.org/) for statistical analyses and data visualization. The limma program was used to examine the differential expression genes (DEGs) of the DDR gene sets between LUSC and normal tissue at *p* < 0.05 with a two‐fold change. The heatmap figure was visualized with “pheatmap” package.

### Risk model construction and validation

Univariate cox regression analysis and least absolute shrinkage and selection operator (LASSO)‐penalized Cox regression analysis (quantile cut‐off = 0.25) were used to identify the DDR‐related prognostic signature. A *p*‐value <0.05 in univariate Cox regression analysis was considered statistically significant, whereas DDR genes were considered eligible for LASSO regression analysis only if they showed significance in the Cox regression analysis. Functional analysis of those genes was performed, and their coefficients were determined through the minimum standard. We stratified LUSC patients into high‐risk and low‐risk groups with DDR associated gene‐based risk score prediction model. The formula for calculating the risk score of each LUSC patient was as follows: risk score = (coefficient mRNA1 × expression of messenger RNA1 [mRNA1]) + (coefficient mRNA2 ×expression of mRNA2) + …… + (coefficient mRNAn × expression mRNAn). mRNA indicates the FPKM value of specific gene in TCGA. Patients were then grouped into two datasets, the training dataset and the testing dataset, with the ratio of 7:3. The ROC curve analysis was performed, respectively, in training dataset, testing dataset, and the complete dataset to value the accuracy of the DDR signature. Differences between the high‐ and low‐risk groups were evaluated by the KM curve and log‐rank test. *p*‐Value <0.05 was considered statistically different.

### Functional and pathway enrichment analysis

GO enrichment analysis includes enrichment in cellular components (CC), molecular function (MF), and biological processes (BP).^10^ GO enrichment analysis and enrichment map of differential expressed DDR genes was performed by “clusterProfiler” package. GSEA was performed with GSEA with FDR q‐value < 0.05. Differential regulated genes were analyzed for high‐risk group compared to low‐risk group.

### Relationship between DDR signature and immune infiltration

CIBERSORT was used for appraising the percentage of different types of tumor‐infiltrating immune cells. LM22 signature file was used to analyze the integrated immune cell types.^11^ We used “ggplot2” package in R Studio to visualize the immune infiltration differential between high‐risk and low‐risk groups based on the prediction model.

## RESULT

### DDR gene expression is different in LUSC and normal tissue

**FIGURE 1:**
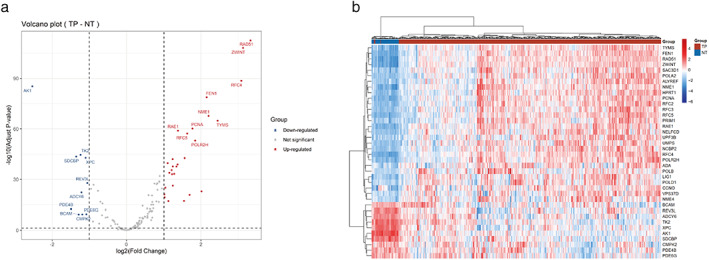
Differentially expressed DDR genes (DDR DEGs) between LUSC and normal tissue. (a) Volcano plots of DDR DEGs. X–axis represents the fold change of gene expression and y‐axis stands for adjusted *p* value. (b) Heatmap of DDR DEGs

DEGs between LUSC and normal tissue were analyzed on 150 DDR genes. In the TCGA database, including 502 LUSC samples and 49 normal lung samples, 28 upregulated DDR genes and 10 downregulated DDR genes of LUSC were identified relative to normal tissue (Figure 1(a); Table S1). Heatmaps show the levels of the differentially expressed genes in detail (Figure 1(b)).

### Construction of DDR‐related gene signature

**FIGURE 2:**
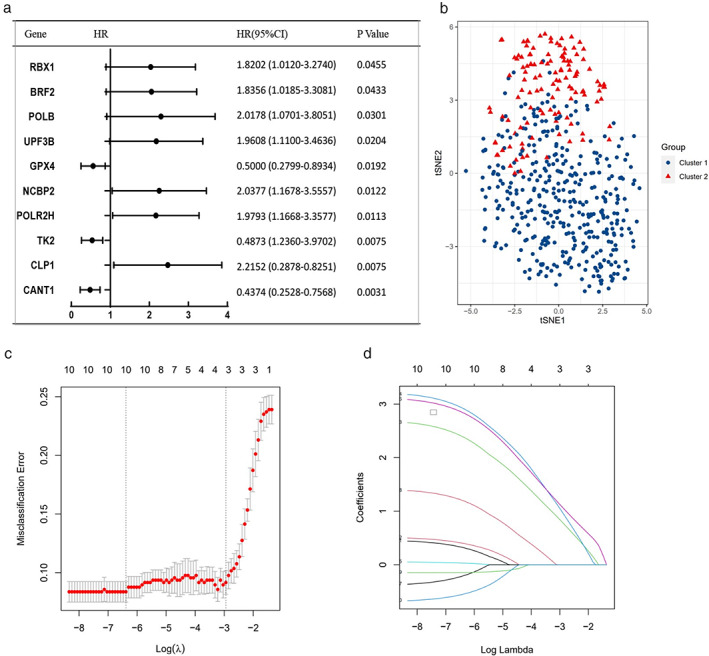
Characterization of DDR related 10 gene signatures. (a) The ten DDR genes filtered with univariance Cox regression. (b) tSNE plot for the cluster 1 (high‐risk group) and cluster 2 (low‐risk group). (c) The LASSO regression analysis, lambda. Min = 0.001684919. (d) Selecting the best parameters for LUSC on the basis of LASSO model

All the DDR genes were screened with univariate Cox regression analysis on only LUSC samples. Ten genes (CLP1, NCBP2, POLB, POLR2H, UPF3B, BRF2, RBX1, GPX4, TK2, and CANT1) were significantly correlated with overall survival (OS) of LUSC (*p* < 0.05) (Figure 2(a); Table S2). We clustered 502 LUSC samples into two groups (cluster 1 and cluster 2, 382 cases and 120 cases, respectively) based on the expression of these 10 DDR genes in TCGA. tSNE plot showed that the two clusters could be classified distinctively (Figure 2(b)).

Further, these 10 DDR genes were measured as predictive genes for LASSO analysis. The optimal value of the lambda penalty parameter defined by performing 10 times‐validations in the training set was selected to obtain the final risk score. All the 10 DDR genes went through the LASSO algorithm were qualified for constructing a risk signature (Figure 2c, d). The risk score formula to predict OS was developed as follows: risk score = 0.31980348* CLP1 + 0.39994951* NCBP2 + 2.38617579* POLB +2.91812372 * POLR2H + 0.03078582* UPF3B + 2.83727830* BRF2 + (−0.20658477)* RBX1 + 1.19580540* GPX4 + (−0.14460181)*TK2 + (−0.54947184)* CANT1. The seven high risk genes CLP1, NCBP2, POLB, POLR2H, UPF3B, BRF2, and GPX4 and three low risk genes RBX1, TK2, and CANT1 have their matched coefficient number.

### Prognostic value of DDR‐related gene signature

**FIGURE 3:**
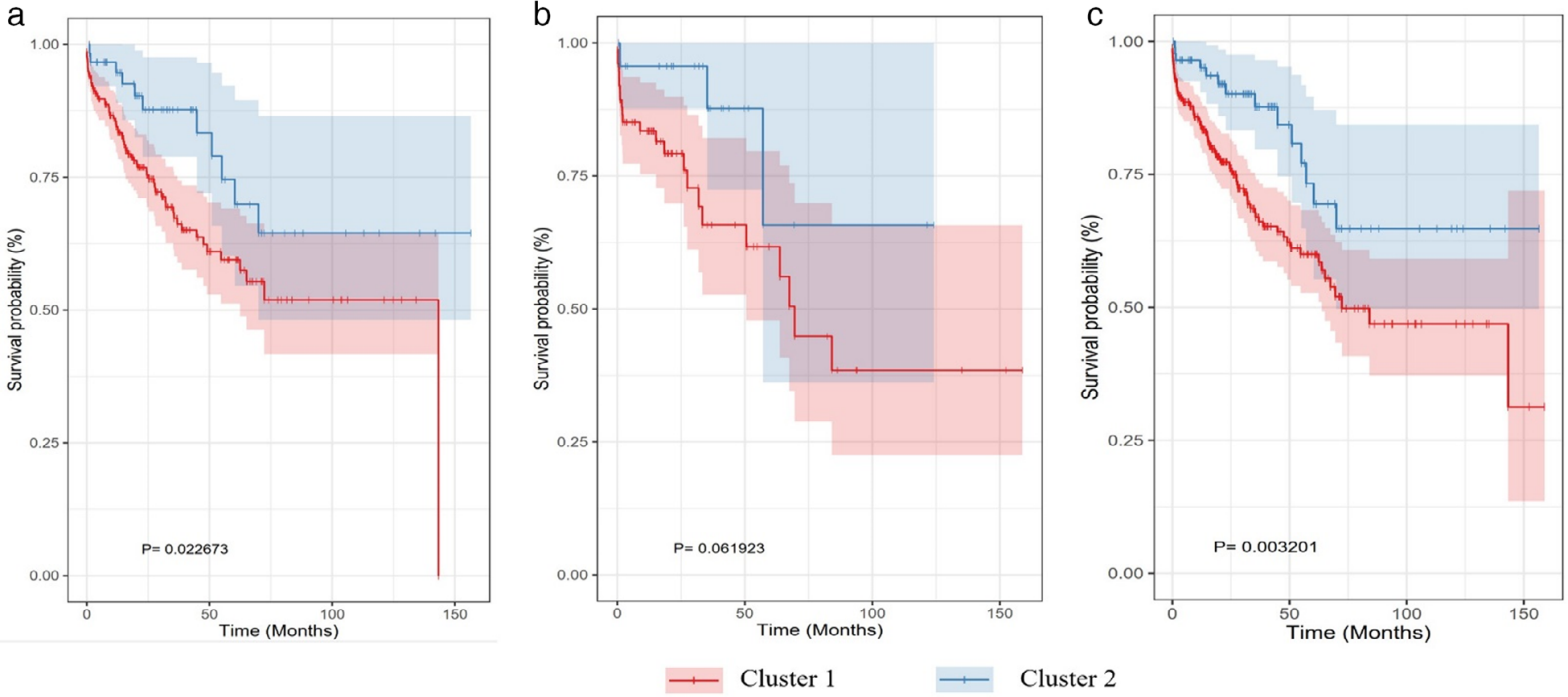
Kaplan–Meier curve analysis of overall survival between high‐ and low‐risk groups. (a) The training dataset; (b) testing dataset; (c) complete dataset

**FIGURE 4:**
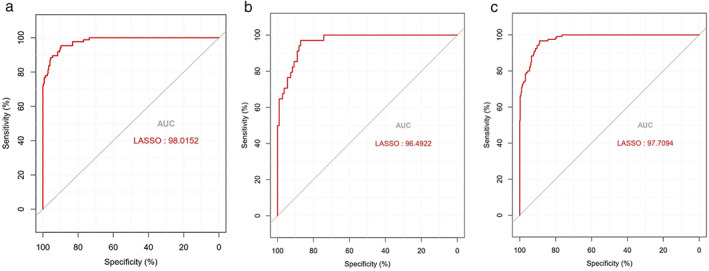
The multiple ROC curve for the DDR‐related gene signature. (a) The training dataset; (b) testing dataset; (c) complete dataset

**FIGURE 5:**
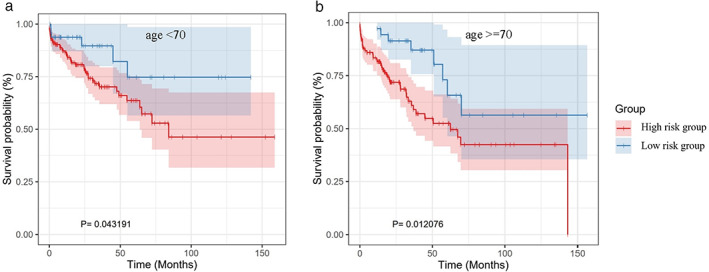
Kaplan–Meier survival curves for the high‐risk and low‐risk groups stratified by age. For both age = 70 (B) groups, patients in the high risk group tended to have a worse overall survival rate compared to the low risk group

A worse prognosis of LUSC patients was observed with cluster 1 (high‐risk group) than patients with cluster 2 (low‐risk group). The patients’ background data of the two risk groups were listed in Table 1. The KM curve analysis showed that the low‐risk group had a better prognosis than the high‐risk group in the training set (*p* = 0.022673) (Figure 3(a)) and complete set (*p* = 0.003201) (Figure 3(c)). However, because of the small numbers in one cluster, the KM curve of testing set did not meet the significance level (Figure 3(b)). Further, the AUC, which is defined as the area under ROC curve, was 0.98, 0.96, and 0.97 in the training dataset, the testing dataset, and the complete dataset, respectively, implying that the DDR‐related 10‐genes signature has good accuracy in the prognostic prediction of LUSC (Figure 4(a)–(c)). We also inspected the prognostic value of the DDR model stratified by age. For both age <70 and age ≥70 groups, patients in the high‐risk group tended to have a worse OS (Figure 5(a),(b).

**TABLE 1:** Baseline data for patients after grouping by DDR‐related signature

	Level	High‐risk	Low‐risk	*p*
No.		382	120	
Group (%)	High‐risk	382 (100.0)	0 (0.0)	<0.001
	Low‐risk	0 (0.0)	120 (100.0)	
Time, median [IQR]		618.00 [239.00, 1168.50]	925.00 [418.50, 1568.50]	0.011
Vital status (%)	Alive	210 (55.0)	75 (62.5)	0.17
	Dead	172 (45.0)	45 (37.5)	
Gender (%)	Female	99 (25.9)	32 (26.7)	0.965
	Male	283 (74.1)	88 (73.3)	
Age, mean (SD)		67.30 (8.67)	66.80 (8.36)	0.582
Intermediate dimension, median [IQR]		0.80 [0.60, 1.00]	0.70 [0.60, 1.00]	0.461
TNM stage (%)	No.	3 (0.8)	1 (0.8)	0.097
	Stage I	186 (48.7)	59 (49.2)	
	Stage II	131 (34.3)	31 (25.8)	
	Stage III	59 (15.4)	25 (20.8)	
	Stage IV	3 (0.8)	4 (3.3)	
T (%)	T1	90 (23.6)	24 (20.0)	0.123
	T2	219 (57.3)	75 (62.5)	
	T3	59 (15.4)	12 (10.0)	
	T4	14 (3.7)	9 (7.5)	
N (%)	N0	242 (63.4)	78 (65.0)	0.399
	N1	103 (27.0)	28 (23.3)	
	N2	27 (7.1)	13 (10.8)	
	N3	4 (1.0)	1 (0.8)	
	NX	6 (1.6)	0 (0.0)	
M (%)	M0	306 (81.0)	106 (88.3)	0.01
	M1	2 (0.5)	3 (2.5)	
	M1a	0 (0.0)	1 (0.8)	
	M1b	1 (0.3)	0 (0.0)	
	MX	69 (18.3)	10 (8.3)	
Pack, years smoked (%)		64 (16.8)	14 (11.7)	0.376
	≤30	81 (21.2)	25 (20.8)	
	>30	237 (62.0)	81 (67.5)	
Race (%)	Asian	7 (2.3)	2 (2.3)	0.447
	Black or African American	26 (8.6)	4 (4.5)	
	White	268 (89.0)	82 (93.2)	
Ethnicity (%)	Hispanic or Latino	5 (2.0)	3 (4.2)	0.53
	Non‐Hispanic or Latino	248 (98.0)	69 (95.8)	

### Relationship between the gene signature and immune cell

**FIGURE 6:**
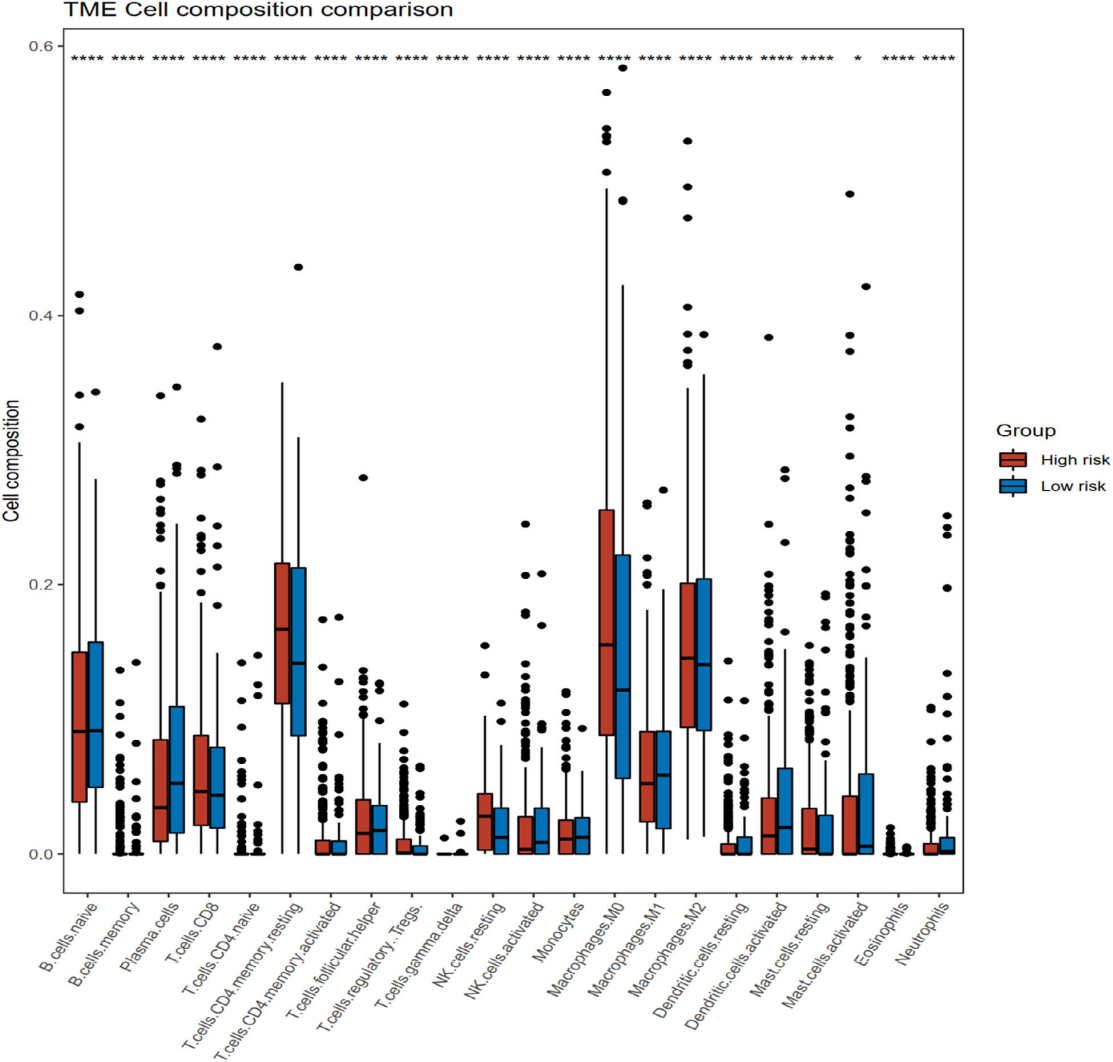
Barplot of 22 immune cells content in the high‐risk and low‐risk group

LUSC samples in the TCGA database showed a difference for all the 22 immune cell types between the high‐ and low‐risk groups (Figure 6). Cell types, of which compositions were slightly higher in high‐risk group, were naive B cells, plasma cells, memory‐resting CD4^+^ T cells, nd T cells, activated natual killer (NK) cells, monocytes, M1 macrophages, resting dendritic cells, activated dendritic cells, and neutrophils. The tumor microenvironment in the low‐risk group was infiltrated with more memory B cells, CD8^+^ T cells, memory‐activated CD4^+^ T cells, follicular helper T cells, regulatory T cells, resting NK, M0 macrophages, M2 macrophages, and resting mast cells.

### Enrichment analyses of DEGs based on the clustering

**FIGURE 7:**
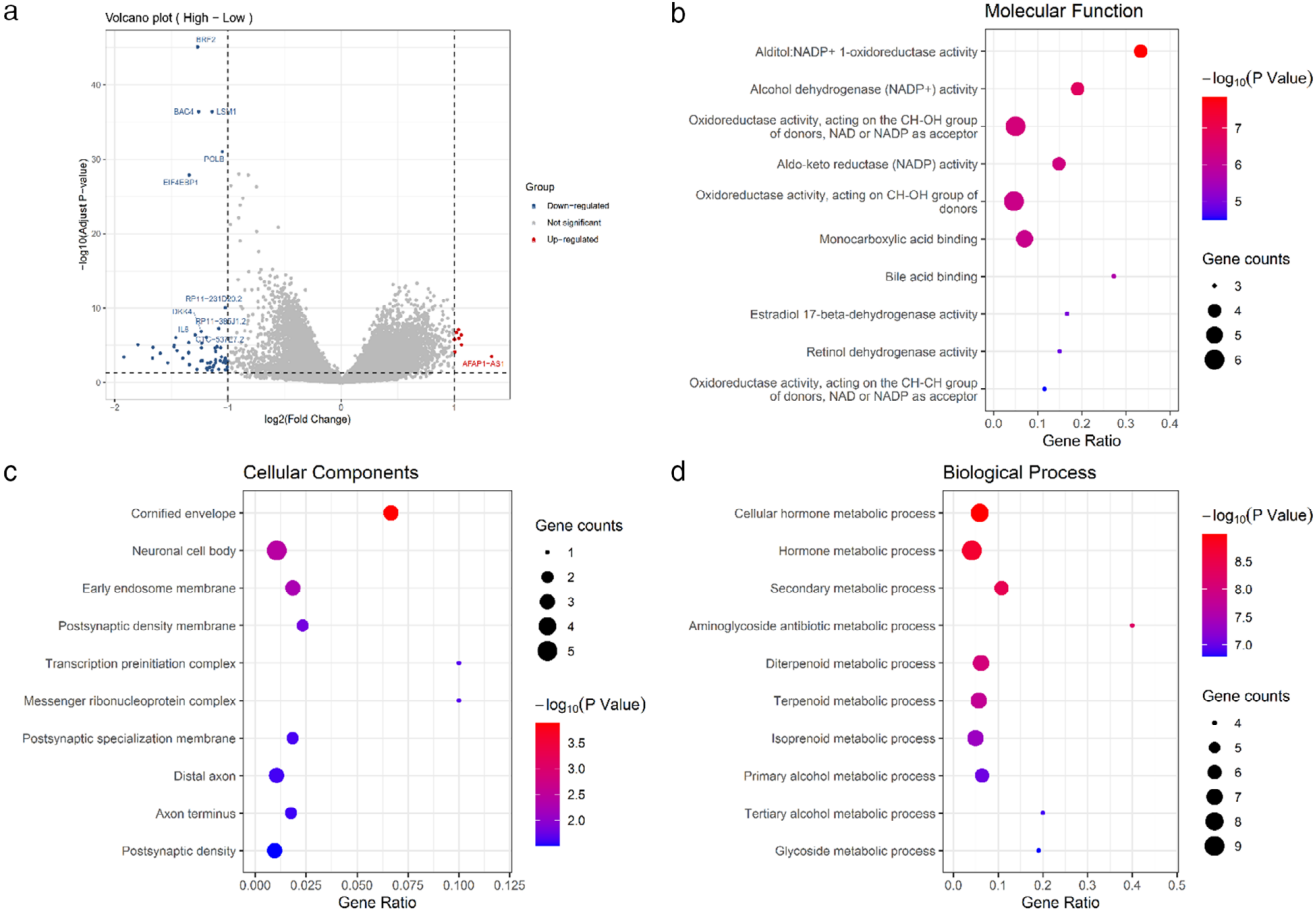
The DEGs based on the clustering with DDR genes signature. (a) Volcano plots of DEGs. (b) GO enrichment of DEGs in molecular function; (c) GO enrichment of DEGs in cell component; (d) GO enrichment of DEGs in biological process

Based on the results of DDR signature clustering, there are 58 downregulated genes and eight upregulated genes with fold change >2 in the high‐risk group (Figure 7(a); Table S3). GO term enrichment analysis demonstrated that the DEGs were principally enriched in NADP^+^ 1‐oxidoreductase activity in molecular function (Figure 7(b)). Cell component analysis indicated that genes were significantly enriched in cornified envelope (Figure 7(c)). Biological process analysis demonstrated that the genes were principally involved in the cellular hormone metabolic process (Figure 7(d)).

**FIGURE 8:**
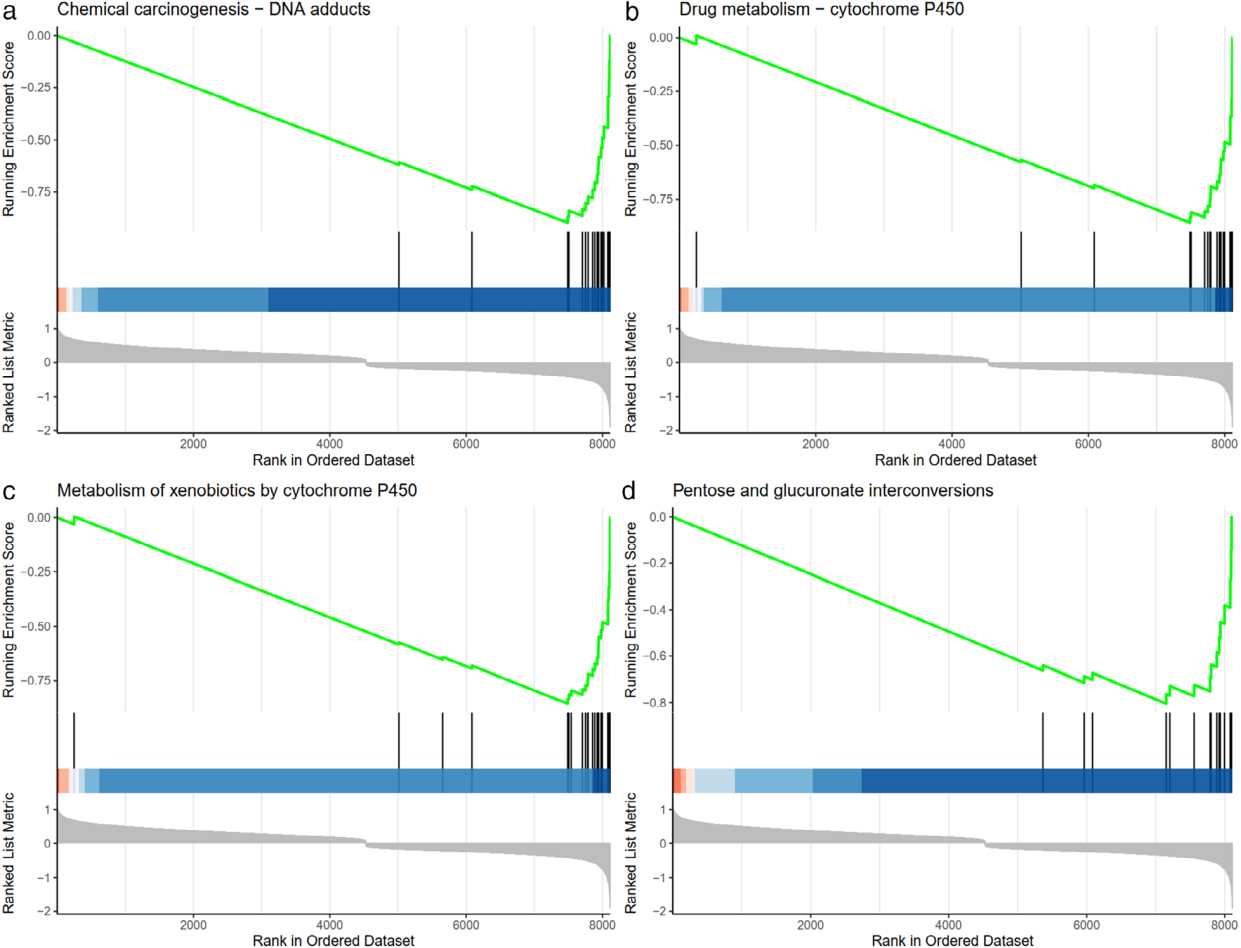
GSEA analysis of the enrichment pathways for DEGs between high‐ and low‐risk groups. In ascending order of enrichment score, the top 4 potential pathways are (a) chemical carcinogenesis‐DNA adducts, (b) drug metabolism cytochrome P450, (c) metabolism of xenobiotics by cytochrome P450 and (d) pentose and glucuronate interconversions

The potential pathways or functions of DDR‐related genes signature were explored by performing a GSEA. As shown in Figure 8(a)–(d), we discovered that patients in the high‐risk group relative to low‐risk group were mainly involved in DNA adducts, drug metabolism‐cytochrome P450, metabolism of xenobiotics by cytochrome P450, and pentose and glucuronate interconversions.

## DISCUSSION

DNA damage occurs through exogenous and endogenous processes. Carcinogens can reduce the DNA damage through myriad mechanisms. As a pivotal mechanism to preserve genome stability and repair DNA lesions, deficiency in the DNA repair pathway might give rise to hypersensitivity to carcinogens and the accumulation of DNA lesions influencing tumor development, metastasis, and prognosis.^12^

In this study, we systematically investigate the role of the DDR gene in the prognosis of LUSC. By conducting a univariate Cox regression screen and performing LASSO‐penalized regression analysis, we successfully formed a 10 DDR genes signature. The KM curve showed that this DDR signature could stratify patients' OS efficiently. ROC analysis results verified that the DDR signature had a high accuracy in predicting the OS of LUSC and had a good prognostic value.

Previous studies suggested that germline mutations in DNA repair genes increase the predisposition to lung cancer.^13^ Supporting this, it has been shown that in ~2.5% of all cancer, a germline mutation in a DNA repair gene was associated with cancer development, including LUSC.^14^ The other way round, LUSC generally exhibits relatively a higher somatic mutation frequency in contrast with different tumor types.^15^ A high proportion occurred in genes that are involved in the maintenance of genome integrity through chromatin modification and DNA damage response and repair and specific DDR gene alterations tend to associate with worse progression‐free survival to initial chemotherapy.^16^ For diagnosis and treatment, it is necessary to investigate further the predictive value resulting in the development of LUSC.

Among these 10 DDR genes in the predictive model, POLB, POLR2H, and BRF2 exhibit the highest coefficient (coefficient >2/<−2). POLB is one of the components of BER. PARP1 has been a popular target for targeted therapies in the past few years, of which role in BER is detecting single‐strand breaks. PARP1 works as a signal for the repair machinery, recruiting the scaffold protein XRCC1, LIG3, and POLB,^17^ to complete repair. The carrier status for the POLB rs3136797 germline mutation is associated with a worse prognosis for lung cancer and demonstrates poor sensitivity to cisplatin treatment.^18^ POLR2H, the necessary subunit of RNA polymerase II, was essential for the transcription of DNA.^19^ Du et al.^20^ indicated that POLR2H expression correlates with the occurrence and progression of prostate cancer. TFIIIB is a known target of regulation by oncogenes and tumor suppressors. TFIIIB‐mediated transcription is downregulated in a variety of cancers. BRF2, a component of TFIIIB required for gene external RNA pol III transcriptions, was identified as an oncogene in LUSC through integrative genomic analysis.^21^ The related study of BRF2 was extremely rare. One study used the Oncomine database to study the expression of BF2 in tumors in a subset of the patient samples and showed that BRF2 is both over‐ and under‐expressed in lung cancer.

We also investigated the correlation between DDR signature and the clinical factors in present study. In our study, DDR signature independently predict the OS of LUSC without the need to consider whether it is advanced age. In addition, the GSEA result revealed that patients in the high‐risk group were negatively enriched in DNA adducts, drug metabolism‐cytochrome P450, metabolism of xenobiotics by cytochrome P450, and pentose and glucuronate interconversions.

It is reported that carcinogens may fall into two categories^22^: activation‐dependent (e.g., polycyclic aromatic hydrocarbons) and activation‐independent (e.g., ultraviolet and ionizing radiation).^23^, ^24^ Activation‐dependent carcinogens require metabolic activation or molecular modification in host cells to transform them into reactive intermediates or carcinogenic metabolites. The reactions, including oxidation, reduction, or hydrolysis, mainly involve cytochrome P450 (CYP) mixed function oxidase isoforms. Bulky chemical adducts are commonly formed as a result of the interaction between activated carcinogens and DNA. Accordingly, our prognostic signature classified by 10 DDR genes showed that compared to low‐risk group, the DEGs of high‐risk group enrich in chemical adduct and P450 related pathway, implicating relatively an indirect DNA damage correlation.

## CONCLUSION

In conclusion, our study identified DDR‐related signature that could be involved in the prognosis of LUSC. To our knowledge, this is the first study that developed a DDR‐associated risk model in LUSC. In addition, our study also investigated the potential relationship between DDR and clinical data, and DNA damage repair and immune cells. The findings of this study provide new insights to understanding the role of DNA damage repair in LUSC and the prediction obtained from the bioinformatics analysis can be verified by future experimental studies.

## CONFLICT OF INTEREST

The authors declare no conflicts of interest.

## Supporting information


**Table S1** Differential expressed DDR genes between LUSC and normal tissue.
**Table S2** DDR genes analyzed by Cox regression on LUSC samples.
**Table S3** DEGs between high‐ and low‐risk groups.

## Data Availability

The datasets generated during the current study are available in the TCGA database (https://portal.gdc.cancer.gov/).

## References

[1] Sung H , Ferlay J , Siegel RL , Laversanne M , Soerjomataram I , Jemal A , et al. Global cancer statistics 2020: GLOBOCAN estimates of incidence and mortality worldwide for 36 cancers in 185 countries. CA Cancer J Clin. 2021;71:209–49. 33538338 10.3322/caac.21660

[2] Herbst RS , Morgensztern D , Boshoff C . The biology and management of non‐small cell lung cancer. Nature. 2018;553:446–54. 29364287 10.1038/nature25183

[3] Langer CJ , Obasaju C , Bunn P , Bonomi P , Gandara D , Hirsch FR , et al. Incremental innovation and Progress in advanced squamous cell lung cancer: current status and future impact of treatment. J Thorac Oncol. 2016;11:2066–81. 27575423 10.1016/j.jtho.2016.08.138

[4] Hirsch FR , Varella‐Garcia M , Bunn PA Jr , di Maria MV , Veve R , Bremnes RM , et al. Epidermal growth factor receptor in non‐small‐cell lung carcinomas: correlation between gene copy number and protein expression and impact on prognosis. J Clin Oncol. 2003;21:3798–807. 12953099 10.1200/JCO.2003.11.069

[5] Cancer Genome Atlas Research N . Comprehensive genomic characterization of squamous cell lung cancers. Nature. 2012;489:519–25. 22960745 10.1038/nature11404 PMC3466113

[6] Drilon A , Rekhtman N , Ladanyi M , Paik P . Squamous‐cell carcinomas of the lung: emerging biology, controversies, and the promise of targeted therapy. Lancet Oncol. 2012;13:e418–26. 23026827 10.1016/S1470-2045(12)70291-7

[7] Jamal‐Hanjani M , Wilson GA , McGranahan N , Birkbak NJ , Watkins TBK , Veeriah S , et al. Tracking the evolution of non‐small‐cell lung cancer. N Engl J Med. 2017;376:2109–21. 28445112 10.1056/NEJMoa1616288

[8] Burgess JT , Rose M , Boucher D , Plowman J , Molloy C , Fisher M , et al. The therapeutic potential of DNA damage repair pathways and genomic stability in lung cancer. Front Oncol. 2020;10:1256. 32850380 10.3389/fonc.2020.01256 PMC7399071

[9] Ragu S , Matos‐Rodrigues G , Lopez BS . Replication stress, DNA damage, inflammatory cytokines and innate immune response. Genes (Basel). 2020;11(4):409. 10.3390/genes11040409 PMC7230342 32283785

[10] Harris MA , Clark J , Ireland A , Lomax J , Ashburner M , Foulger R , et al. The gene ontology (GO) database and informatics resource. Nucleic Acids Res. 2004;32:D258–61. 14681407 10.1093/nar/gkh036 PMC308770

[11] Newman AM , Liu CL , Green MR , Gentles AJ , Feng W , Xu Y , et al. Robust enumeration of cell subsets from tissue expression profiles. Nat Methods. 2015;12:453–7. 25822800 10.1038/nmeth.3337 PMC4739640

[12] Li W , Zhang M , Huang C , Meng J , Yin X , Sun G . Genetic variants of DNA repair pathway genes on lung cancer risk. Pathol Res Pract. 2019;215:152548. 31337555 10.1016/j.prp.2019.152548

[13] Parry EM , Gable DL , Stanley SE , Khalil SE , Antonescu V , Florea L , et al. Germline mutations in DNA repair genes in lung adenocarcinoma. J Thorac Oncol. 2017;12:1673–8. 28843361 10.1016/j.jtho.2017.08.011 PMC5659909

[14] Knijnenburg TA , Wang L , Zimmermann MT , et al. Genomic and molecular landscape of DNA damage repair deficiency across the cancer genome atlas. Cell Rep. 2018;23:239–54. e6. 29617664 10.1016/j.celrep.2018.03.076 PMC5961503

[15] Kandoth C , McLellan MD , Vandin F , et al. Mutational landscape and significance across 12 major cancer types. Nature. 2013;502:333–9. 24132290 10.1038/nature12634 PMC3927368

[16] Dai J , Jiang M , He K , Wang H , Chen P , Guo H , et al. DNA damage response and repair gene alterations increase tumor mutational burden and promote poor prognosis of advanced lung cancer. Front Oncol. 2021;11:708294. 34604048 10.3389/fonc.2021.708294 PMC8479169

[17] Tomasova K , Cumova A , Seborova K , Horak J , Koucka K , Vodickova L , et al. DNA repair and ovarian carcinogenesis: impact on risk, prognosis and therapy outcome. Cancers (Basel). 2020;12:1713. 10.3390/cancers12071713 PMC7408288 32605254

[18] Nemec AA , Abriola L , Merkel JS , de Stanchina E , DeVeaux M , Zelterman D , et al. DNA polymerase Beta germline variant confers cellular response to cisplatin therapy. Mol Cancer Res. 2017;15:269–80. 28074003 10.1158/1541-7786.MCR-16-0227-T PMC5334281

[19] Du YJ , Hou YL , Hou WR . Molecular characterization of a gene POLR2H encoded an essential subunit for RNA polymerase II from the Giant panda (Ailuropoda Melanoleuca). Mol Biol Rep. 2013;40:1495–8. 23070920 10.1007/s11033-012-2192-9

[20] Fan S , Liang Z , Gao Z , Pan Z , Han S , Liu X , et al. Identification of the key genes and pathways in prostate cancer. Oncol Lett. 2018;16:6663–9. 30405806 10.3892/ol.2018.9491 PMC6202544

[21] Cabarcas S , Schramm L . RNA polymerase III transcription in cancer: the BRF2 connection. Mol Cancer. 2011;10:47. 21518452 10.1186/1476-4598-10-47 PMC3098206

[22] Barnes JL , Zubair M , John K , Poirier MC , Martin FL . Carcinogens and DNA damage. Biochem Soc Trans. 2018;46:1213–24. 30287511 10.1042/BST20180519 PMC6195640

[23] Mullenders LHF . Solar UV damage to cellular DNA: from mechanisms to biological effects. Photochem Photobiol. 2018;17:1842–52. 10.1039/c8pp00182k 30065996

[24] Shuryak I , Brenner DJ . Review of quantitative mechanistic models of radiation‐induced non‐targeted effects (Nte). Radiat Prot Dosimetry. 2020;192:236–52. 33395702 10.1093/rpd/ncaa207 PMC7840098

