# Supplementary material for: Identification of a DNA damage repair gene‐related signature for lung squamous cell carcinoma prognosis
Source: Thorac Cancer. 2022 Mar 15;13(8):1143–52. doi: 10.1111/1759-7714.14370 (PMC9013644; doi:10.1111/1759-7714.14370)
Supplement: Supplementary file 1 — Table S1 Differential expressed DDR genes between LUSC and normal tissue. Table S2 DDR genes analyzed by Cox regression on LUSC samples. Table S3 DEGs between high‐ and low‐risk groups. [file TCA-13-1143-s001.docx]

Table 1. Patients’ baseline data after grouped by DDR-related signature

|  | level | High risk | Low risk | p |
| --- | --- | --- | --- | --- |
| n |  | 382 | 120 |  |
| group (%) | High risk | 382 (100.0) | 0 ( 0.0) | <0.001 |
|  | Low risk | 0 ( 0.0) | 120 (100.0) |  |
| time (median [IQR]) |  | 618.00 [239.00, 1168.50] | 925.00 [418.50, 1568.50] | 0.011 |
| vital_status (%) | Alive | 210 ( 55.0) | 75 ( 62.5) | 0.17 |
|  | Dead | 172 ( 45.0) | 45 ( 37.5) |  |
| gender (%) | female | 99 ( 25.9) | 32 ( 26.7) | 0.965 |
|  | male | 283 ( 74.1) | 88 ( 73.3) |  |
| age (mean (SD)) |  | 67.30 (8.67) | 66.80 (8.36) | 0.582 |
| intermediate_dimension (median [IQR]) |  | 0.80 [0.60, 1.00] | 0.70 [0.60, 1.00] | 0.461 |
| TNM.stge (%) | N | 3 ( 0.8) | 1 ( 0.8) | 0.097 |
|  | Stge I | 186 ( 48.7) | 59 ( 49.2) |  |
|  | Stge II | 131 ( 34.3) | 31 ( 25.8) |  |
|  | Stge III | 59 ( 15.4) | 25 ( 20.8) |  |
|  | Stge IV | 3 ( 0.8) | 4 ( 3.3) |  |
| T (%) | T1 | 90 ( 23.6) | 24 ( 20.0) | 0.123 |
|  | T2 | 219 ( 57.3) | 75 ( 62.5) |  |
|  | T3 | 59 ( 15.4) | 12 ( 10.0) |  |
|  | T4 | 14 ( 3.7) | 9 ( 7.5) |  |
| N (%) | N0 | 242 ( 63.4) | 78 ( 65.0) | 0.399 |
|  | N1 | 103 ( 27.0) | 28 ( 23.3) |  |
|  | N2 | 27 ( 7.1) | 13 ( 10.8) |  |
|  | N3 | 4 ( 1.0) | 1 ( 0.8) |  |
|  | NX | 6 ( 1.6) | 0 ( 0.0) |  |
| M (%) | M0 | 306 ( 81.0) | 106 ( 88.3) | 0.01 |
|  | M1 | 2 ( 0.5) | 3 ( 2.5) |  |
|  | M1a | 0 ( 0.0) | 1 ( 0.8) |  |
|  | M1b | 1 ( 0.3) | 0 ( 0.0) |  |
|  | MX | 69 ( 18.3) | 10 ( 8.3) |  |
| pack.years.smoked (%) | | 64 ( 16.8) | 14 ( 11.7) | 0.376 |
|  | <=30 | 81 ( 21.2) | 25 ( 20.8) |  |
|  | >30 | 237 ( 62.0) | 81 ( 67.5) |  |
| race (%) | asian | 7 ( 2.3) | 2 ( 2.3) | 0.447 |
|  | black or african american | 26 ( 8.6) | 4 ( 4.5) |  |
|  | white | 268 ( 89.0) | 82 ( 93.2) |  |
| ethnicity (%) | hispanic or latino | 5 ( 2.0) | 3 ( 4.2) | 0.53 |
|  | not hispanic or latino | 248 ( 98.0) | 69 ( 95.8) |  |
